# *In vivo *breast cancer characterization imaging using two monoclonal antibodies activatably labeled with near infrared fluorophores

**DOI:** 10.1186/bcr3167

**Published:** 2012-04-17

**Authors:** Kohei Sano, Makoto Mitsunaga, Takahito Nakajima, Peter L Choyke, Hisataka Kobayashi

**Affiliations:** 1 Molecular Imaging Program, Center for Cancer Research, National Cancer Institute, NIH, Building 10, RoomB3B69, 10 Center Dr. Bethesda, MD 20892-1088, USA

## Abstract

**Introduction:**

The gene expression profiles of cancer cells are closely related to their aggressiveness and metastatic potential. Antibody-based immunohistochemistry (IHC) of tissue specimens is a common method of identifying expressed proteins in cancer cells and increasingly inform treatment decisions. Molecular imaging is a potential method of performing similar IHC studies *in vivo *without the requirement for biopsy or tumor excision. To date, antibody-based imaging has been limited by high background levels related to slow clearance, making such imaging practical. However, optically activatable imaging agents, which are only fluorescent when bound to their cognate receptor, open the possibility of doing *in vivo *multi-color IHC.

**Methods:**

We describe the use of activatable, near infrared fluorescence-labeled AlexaFluor680 (Alexa680) conjugated panitumumab (Pan) targeted against human epidermal growth factor receptor (EGFR) (Pan-Alexa680) and Indocyanine Green (ICG) conjugated trastuzumab (Tra) targeted against human epidermal growth factor receptor type 2 (HER2) (Tra-ICG) were synthesized and evaluated in cells *in vitro *and in an orthotopic breast cancer mouse model *in vivo*.

**Results:**

Pan-Alexa680 (self-quenched; SQ) and Tra-ICG were initially quenched but demonstrated a 5.2- and 50-fold dequenching capacity under detergent treatment, respectively. *In vitro *microscopy and flow cytometry using MDA-MB-468 (EGFR+/HER2-) and 3T3/HER2 cells (EGFR-/HER2+), demonstrated specific fluorescence signal for each cell type based on binding to Pan-Alexa680(SQ) or Tra-ICG. An *in vivo *imaging study employing a cocktail of Pan-Alexa680(SQ) and Tra-ICG (each 50 μg) was injected into mice with orthotopic MDA-MB-468 and 3T3/HER2 tumors in the breast. Each probe visualized only the target-specific breast tumor.

**Conclusions:**

Multi-color target-specific fluorescence breast cancer imaging can be achieved *in vivo *by employing two activatable fluorescent probes administered as a cocktail. The images allowed us to see a specific receptor expression in each breast tumor without post-image processing.

## Introduction

Antibodies are macromolecules with high binding specificity for target antigens. This makes them excellent scaffolds for molecular imaging probes. However, a central limitation of using antibodies for imaging is that they are slow to clear from the body resulting in non-specific background contamination, reducing the target-to-background ratio. In order to overcome the high background signal found with labeled intact antibodies, labeled antibody fragments, which show more rapid clearance, have been developed. However, faster clearance from the blood pool leads to lower concentrations and, therefore, lower tumor uptake [1].

Optical imaging has many theoretical advantages for tumor identification and characterization. A central advantage is that simultaneous multi-color imaging can be employed to characterize expression profiles [2,3]. By using two or more different antibodies labeled with distinct colors, unbound antibody in the normal tissue can be mathematically subtracted [4,5], since the bio-distribution of antibodies is similar in normal organs and only differs markedly at the tissue expressing the target antigen. However, this approach is somewhat inefficient as it subtracts the target signal as well as the background signal. Therefore, another approach to reducing background signal from two-antibody-based optical is to take advantage of the unique feature of fluorescence that allows it to be quenched or activated depending on its chemical state. Such activatable probes (for example, labeled antibodies) are designed to generate signal only after being bound and processed by the target cancer cells; therefore, high accumulation of antibodies in the target tissue can be clearly visualized while the background signal remains quenched [6,7]. This approach permits many high tumor-to-background ratios to be achieved [8].

In this study, two different antibodies against human epidermal growth factor receptor (EGFR) and human epidermal growth factor receptor type 2 (HER2) were conjugated with activatable near-infrared (NIR) fluorophores, namely Indocyanine Green (ICG) and AlexaFluor680 (Alexa680), which emit light at different wavelengths. We then employed these two activatable antibodies as a cocktail in mice with EGFR and HER2 positive tumors in order to demonstrate the feasibility of this approach.

## Materials and methods

### Reagents

Panitumumab (Pan), a fully human IgG_2 _monoclonal antibody (mAb) directed against the extracellular domain of the human EGFR (HER1), was purchased from Amgen (Thousand Oaks, CA, USA). Trastuzumab (Tra), a recombinant humanized mAb directed against the human HER2, was purchased from Genentech Inc. (South San Francisco, CA, USA). ICG-Sulfo-OSu was purchased from Dojindo Molecular Technologies (Rockville, MD, USA). Alexa680-NHS ester was purchased from Invitrogen Co. (Carlsbad, CA, USA). All other chemicals used were of reagent grade.

### Synthesis of Alexa680 or ICG conjugated antibodies

Panitumumab (0.5 mg, 3.4 nmol) was incubated with Alexa680-NHS ester (39.3 μg, 34 nmol) in 0.1 M Na_2_HPO_4 _(pH 8.6) at room temperature for one hour, followed by the purification with a size exclusion column (PD-10; GE Healthcare, Piscataway, NJ, USA). ICG labeling of trastuzumab was also performed by reacting mAb with ICG at a ratio of 1:6 in the same manner as Pan-Alexa680. The concentrations of Alexa680 and ICG were calculated by measuring the absorption with the UV-Vis system (8453 Value UV-Vis system; Agilent Technologies, Santa Clara, CA, USA) to confirm the number of fluorophore molecules conjugated with each antibody molecule. The protein concentration was also determined by measuring the absorption at 280 nm with a UV-Vis system. The number of Alexa680 and ICG per antibody was adjusted to approximately 4.0 to 4.5 and 0.7 to 1.0, respectively. As a comparison, Pan conjugated with approximately one Alexa680 (always-on type; Pan-Alexa680 (ON)) was also synthesized.

### Determination of quenching capacity *in vitro *

The quenching abilities of each conjugate were investigated by denaturing them with 1% SDS as described previously [9]. Briefly, the conjugates were incubated with 1% sodium dodecyl sulfate (SDS) in PBS for 15 minutes at room temperature. As a control, the samples were incubated in PBS. The fluorescence signal intensity of Pan-Alexa680 was measured with a fluorescence spectrometer (Perkin-Elmer LS55, Perkin-Elmer, Shelton, CT, USA). The change in fluorescence intensity of ICG was investigated with an *in vivo *imaging system (Maestro, CRi Inc., Woburn, MA, USA) using 710 to 760 nm excitation and 800 nm long-pass emission filters.

### Cell culture

The EGFR+/HER2- breast cancer cell line, MDA-MB-468, and the EGFR-/HER2+ cell line, NIH/3T3 (3T3/HER2), were used. Both cell lines were grown in RPMI 1640 (Life Technologies, Gaithersburg, MD, USA) containing 10% fetal bovine serum (Life Technologies), 0.03% L-glutamine, 100 units/mL penicillin, and 100 μg/mL streptomycin in 5% CO_2 _at 37°C.

### Fluorescence microscopy studies

MDA-MB-468 cells or 3T3/HER2 cells (1 × 10^4^) were plated on a covered glass-bottomed culture well and incubated for 16 hours. Pan-Alexa680 (ON or self-quenched; SQ) and Tra-ICG (10 μg/mL) were then added to MDA-MB-468 cells and 3T3/HER2 cells, respectively. The cells were incubated for either one or eight hours followed by washing once with PBS, and fluorescence microscopy was performed using an Olympus BX81 microscope (Olympus America, Inc., Melville, NY, USA) equipped with the following filters: excitation wavelength 590 to 650 nm and 672.5 to 747.5 nm, emission wavelength 662.5 to 747.5 nm and 765 to 855 nm for Alexa680 and ICG, respectively. Transmitted light differential interference contrast images were also acquired. To validate the specific binding of the antibody, excess antibody (100 μg) was used to block 10 μg of dye-antibody conjugates.

### Flow cytometry studies

Fluorescence signal from cells after incubation with Pan-Alexa680 (ON, SQ) or Tra-ICG was measured using a FACS Calibur flow cytometer (BD Biosciences, San Jose, CA, USA) and CellQuest software (BD Biosciences). Briefly, MDA-MB-468 and 3T3/HER2 cells (1 × 10^5^) were incubated with Pan-Alexa680 (ON or SQ) and Tra-ICG at 37°C for one or eight hours, respectively. To validate the specific binding of the antibody, excess antibody (50 μg) was used to block 0.5 μg of dye-antibody conjugates.

### Tumor model

All procedures were carried out in compliance with the Guide for the Care and Use of Laboratory Animal Resources (1996), National Research Council, and approved by the National Cancer Institute Animal Care and Use Committee. Six- to eight-week-old female homozygote athymic nude mice were purchased from Charles River (NCI-Frederick, Frederick, MD, USA). During the procedure, mice were anesthetized with isoflurane. MDA-MB-468 cells (2 × 10^6 ^cells) were injected subcutaneously into the right mammary pads of the mice, and two weeks later (because they grow faster), 3T3/HER2 cells (2 × 10^6 ^cells) were injected into the left mammary pads. The experiments were conducted at six days after 3T3/HER2 cell injection.

### *In vivo *two-color two-activatable imaging targeted for EGFR and HER2

A mixture of Tra-ICG and Pan-Alexa680(SQ or ON) (each 50 μg) was injected via the tail vein into tumor-bearing mice (MDA-MB-468 and 3T3/HER2). The mice were anesthetized with intraperitoneally administered 10% sodium pentobarbital, and fluorescence images were obtained for four days with a flurescence camera (Pearl Imager LI-COR Biosciences using the 700 and 800 nm fluorescence channel or Maestro *in vivo *Imaging System CRi) using two filter sets). The red filter sets were used to image Alexa680 fluorescence and the NIR filter sets were used for ICG fluorescence. The red filter set uses a band-pass filter, which ranges between 615 to 665 nm (excitation) and a long-pass filter over 700 nm (emission); the NIR filter set uses a band-pass filter from 710 to 760 nm (excitation) and a long-pass filter over 800 nm (emission). The tunable emission filter was automatically stepped in 10 nm increments from 650 to 950 nm for the red and NIR filter sets at constant exposure. The spectral fluorescence images consist of autofluorescence spectra and the spectra from Alexa680 and ICG, which were then unmixed, based on their spectral patterns using commercial software (Maestro software; CRi). Mice were sacrificed with carbon dioxide immediately after *in vivo *imaging. The tumors were excised and *ex vivo *imaging was performed using Pearl Imager and Maestro *in vivo *imaging system.

### Statistical analysis

Quantitative data were expressed as mean ± SD. Means were compared using two-way factorial ANOVA followed by the Tukey-Kramer test. *P-*values of < 0.05 were considered statistically significant.

## Results

### Quenching capacity of dye conjugated antibody

The quenching capacities measured by adding 1% SDS to dye-conjugated antibody were 50-, 5.2- and 1.2-fold for Tra-ICG, Pan-Alexa680(SQ), and Pan-Alexa680(ON), respectively.

### *In vitro *fluorescent characterization of probes

**Figure 1:**
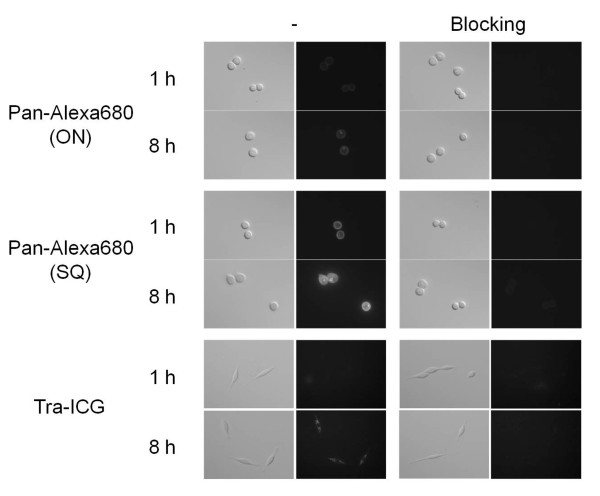
**Fluorescence microscopy studies**. MDA-MB-468 cells and 3T3/HER2 cells were incubated with Pan-Alexa680(ON or SQ) and Tra-ICG, respectively, for one or eight hours. The higher fluorescent signals was detected by Pan-Alexa680(SQ) especially within the tumor cells compared with Pan-Alexa680(ON) after eight hours incubation. Many bright foci could be detected by Tra-ICG at eight hours within the 3T3/HER2 cells. These signals were completely blocked by the addition of excess panitumumab or trastuzumab.

In the microscopy studies (Figure 1), the fluorescence signals from Pan-Alexa680(ON) and Pan-Alexa680(SQ) were observed on the surface of MDA-MB-468 cells one hour after incubation. Both probes also showed fluorescent signal within the cells eight hours after incubation, and the fluorescent dots were brighter for Pan-Alexa680(SQ) than for Pan-Alexa680(ON). These signals were completely blocked by the addition of excess panitumumab. Similarly, fluorescence signal from Tra-ICG was observed within the 3T3/HER2 cells. While signal was minimal at one hour after incubation because of greater quenching magnitude of Tra-ICG than Pan-Alexa680, many bright intracellular foci were present by eight hours, and these signals were blocked by excess trastuzumab.

**Figure 2:**
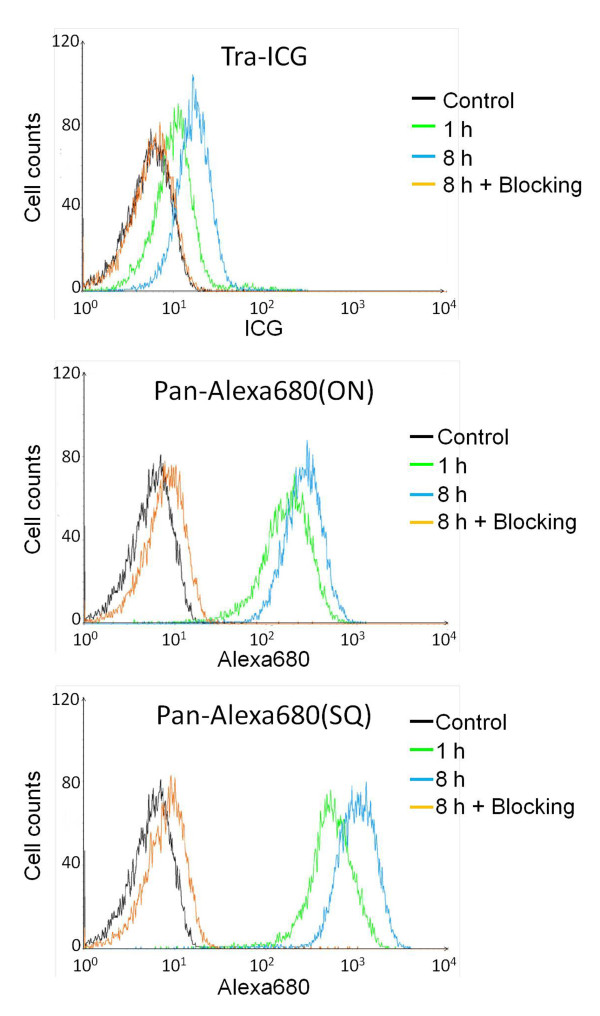
**Flow cytometry studies**. Flow cytometry studies showed specific binding of Pan-Alexa680(ON or SQ) and Tra-ICG to EGFR and HER2, respectively. Pan-Alexa680(SQ) showed brighter signals and increased fluorescent signals over time compared with Pan-Alexa680(ON).

FACS studies were similar to microscopy studies, that is, Pan-Alexa680(ON, SQ) and Tra-ICG exhibited specific binding to MDA-MB-468 and 3T3/HER2 cells, respectively (Figure 2). Pan*-*Alexa680(SQ) showed progressively brighter signal over time compared with Pan-Alexa680(ON) where the signal was lower and constant.

### *In vivo *two-color activatable imaging targeted for EGFR and HER2

**Figure 3:**
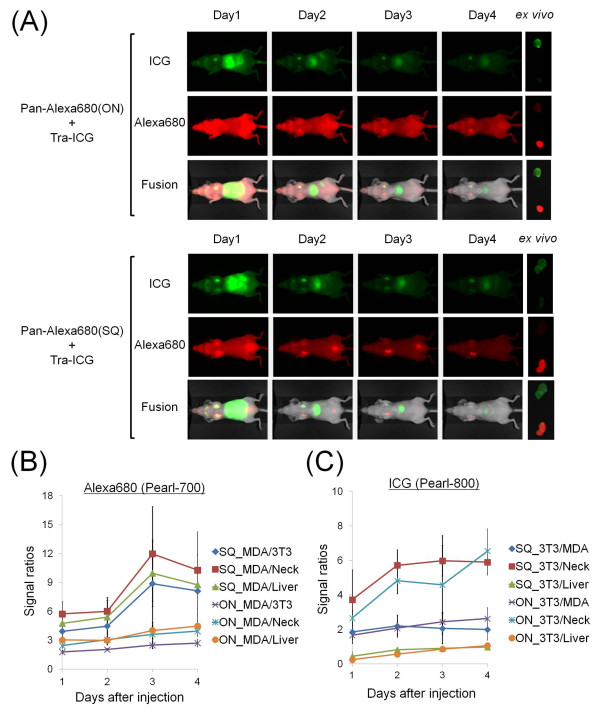
***In vivo *fluorescence imaging studies without post-image processing**. **(A) ***In vivo *and *ex vivo *fluorescence images with mice bearing 3T3/HER2 (left breast) and MDA-MB-468 (right breast) tumors using the Pearl imager which is based on filter sets. The mixture of Pan-Alexa680(ON or SQ) and Tra-ICG was injected via the tail vein. Tra-ICG clearly visualized HER2 expressing 3T3/HER2 tumors. While Pan-Alexa680(SQ) specifically detected EGFR expressing MDA-MB-468 tumors with higher contrast than Pan-Alexa680(ON). **(B, C) **The tumor to non-targeted tumor, neck, and liver ratios for Alexa680 (B) or ICG (C) fluorescence intensity are displayed. Data are represented as mean ± s.d. (*n *= 4 mice).

**Figure 4:**
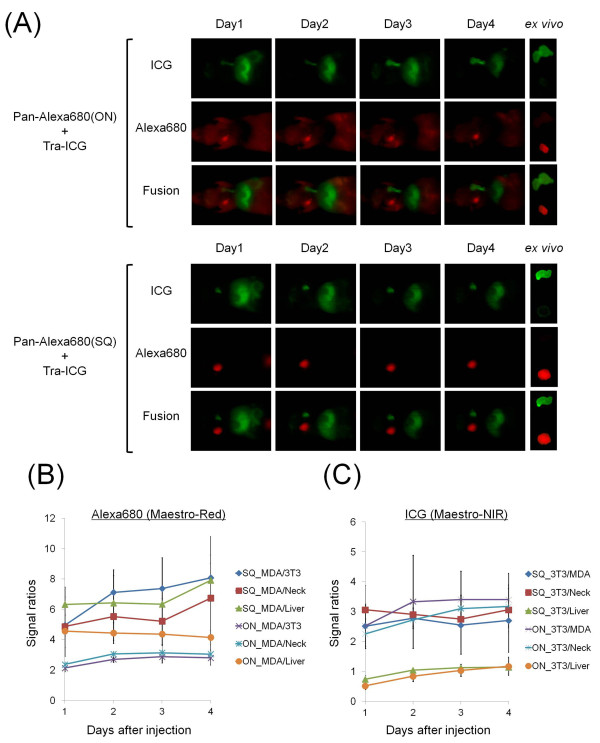
***In vivo *fluorescence imaging studies with post-image processing using the spectral separation**. **(A) ***In vivo *and *ex vivo *fluorescence images of mice bearing 3T3/HER2 (left breast) or MDA-MB-468 (right breast) tumors as depicted by the Maestro imager, which employs an incremental filter and unmixing algorithms. The Alexa680 spectrum images shows a target specific image with Pan-Alexa680(SQ). In contrast, high background signal was observed for Pan-Alexa680(ON). Tra-ICG clearly visualized HER2 expressing 3T3/HER2 tumors in both groups. **(B, C) **The tumor to non-targeted tumor, neck and liver ratios for Alexa680 (B) or ICG (C) fluorescence intensity are depicted. Data are represented as mean ± s.d. (*n *= 4 mice).

Figure 3A shows the images of tumor-bearing mice (MDA-MB-468 and 3T3/HER2) injected with the mixture of Pan-Alexa680(ON or SQ) and Tra-ICG, which was obtained with the Pearl Imager. Pan-Alexa680(ON) detected the targeted tumor (MDA-MB-468, EGFR+); however, high background and non-specific tumor uptake (3T3/HER2) are evident on Day 1 through Day 3 post injection. At Day 4, relatively selective images of EGFR positive (MDA-MB-468) tumors were obtained. In contrast, Pan-Alexa680(SQ) clearly visualized MDA-MB-468 with high tumor-to-background ratios as early as Day 2 and at all later time points, although a slight signal was detected in the 3T3/HER2 tumor and bladder at Day 1. On the other hand, Tra-ICG specifically visualized the HER2 positive 3T3/HER2 tumors, although slight fluorescent signals were noticed in the liver and intestine. Tumor-to-background (non-targeted tumors, liver, and neck) ratios were significantly higher (*P *< 0.01) for Pan-Alexa680(SQ) compared with Pan-Alexa680(ON) for four days (Figure 3B). Tumor-to-non-targeted tumor, tumor-liver and tumor-neck ratios were 8.1, 10.3, and 8.8 for Pan-Alexa680(SQ) at Day 4; whereas, they were 2.7, 3.9, and 4.5 for Pan-Alexa680(ON). In contrast, tumor-to-background ratios with Tra-ICG were not significantly different between the two groups (Figure 3C). The Maestro spectral fluorescence imager provided similar images to the Pearl imager (Figure 4). For instance, tumor-to-non-targeted tumor, tumor-liver and tumor-neck were 8.1, 6.7, and 7.9 for Pan-Alexa680(SQ) at Day 4, whereas, these same ratios were 2.8, 3.0, and 4.2 for Pan-Alexa680(ON). Tumor-to-background ratios on Tra-ICG were not significantly different between both groups.

## Discussion

Activatable optical imaging using a labeled targeting antibody can be a sensitive and specific method of performing *in vivo *molecular imaging with high tumor-to-background ratios [6]. Like immunohistochemistry, each antibody can be labeled with a different type of activatable fluorophore allowing multi-color imaging. In this case, two different EGFR receptors (EGFR and HER2) were targeted successfully.

Activatable fluorescence probes can be based on one of several mechanisms, including self-quenching (Homo-FRET), auto-quenching (Hetero-FRET), H-dimer formation, photo-induced electron transfer (PeT) and uncaging [6]. Among these, self-quenching and auto-quenching fluorophores are relatively easy to synthesize. Both antibody conjugates showed intense bright fluorescence foci within tumor cells expressing the respective targets, suggesting internalization of the antibody conjugate was required for activation [7,10].

In the case of the two activatable antibody conjugates described here, Tra-ICG and Pan-Alexa680(SQ), we showed that they could achieve impressive dequenching ratios of 50- and 5.2-fold respectively. The difference of dequenching capacity for ICG and Alexa680 can be explained by distinct quenching mechanisms of two conjugates. The fluorescence of ICG can be quenched by auto-quenching that is hetero-FRET between ICG and aromatic amino acids of antibodies; in contrast, the fluorescence of Alexa680 can be quenched by homo-FRET between two or more Alexa680 molecules in a conjugate. Therefore, greater numbers of Alexa680 on a single antibody could achieve better quenching of Pan-Alexa680(SQ). However, the bio-distribution was dramatically changed and showed high uptake in the liver, when seven or more Alexa680s were conjugated with a single panitumumab. Thus, the number of Alexa680 molecules conjugated with a single antibody had to be limited up to five despite of incomplete self-quenching of Pan-Alexa(SQ). Therefore, fluorescence signal of activated Pan-Alexa680(SQ) was greater; however, the dequenching ratios of Pan-Alexa(SQ) were lower than that of Tra-ICG.

We employed two imaging devices in this study. The Pearl imager (Pearl, LI-COR Biosciences) can detect two kinds of NIR probes (emission wavelength 700 and 800 nm) separately by using optical filter sets and a highly sensitive CCD camera. However, the multispectral fluorescence imager (Maestro, CRi) is equipped with a tunable crystal filter that can distinguish between two or more distinct fluorescence signals emanating from different external and internal fluorophores by their spectral separation [11]. The former system can simply show photon counts at the specific range of wavelength, while the latter system can extract the spectral signal from each distinct fluorophores. As shown in the results, spectral unmixing is able to separate the different colors than optical filtering, allowing multi-targeted color images with high tumor-to-background ratios.

In contrast to previous studies, which employed subcutaneous xenografts, we employed orthotopic bilateral breast cancer tumor models, each one of which expressed either EGFR or HER2. This enabled us to use the orthotopic tumors as controls for each other [12]. A potential alternative will be the use of fluorescent proteins, which are excellent endogenous fluorescence emitters to be used as a powerful tool for depicting various biological processes both *in vitro *and *in vivo *[13]. However, for the medical application, fluorescence proteins require *in vivo *virus-mediated gene transfection [14,15], which is unlikely to be permitted for the diagnosis in humans at least in the near term.

Since tumors show genomic and proteomic diversity, expression profiles vary widely even within a single lesion. EGFR and HER2 play an important role in carcinogenesis; overexpression of one or more members of the EGFR family has been shown in a number of malignancies, including breast, ovarian, non-small cell lung cancer and squamous cell carcinomas. Furthermore, high levels of expression relates to a poor prognosis [16-18]. However, currently it is necessary to extract tissue and stain using immunohistochemistry methods. Detecting expression levels of EGFR profile *in vivo *could be helpful for detecting and characterizing malignant tumors, as well as determining therapy.

## Conclusion

By employing two-color, activatable fluorophores conjugated to specific antibodies, multi-target specific fluorescence imaging could be achieved with high target-to-background ratios that allowed us to see a specific receptor expression in each breast tumor without post-processing images. Such methods may be useful in the future for *in vivo *characterization of breast cancers.

## Abbreviations

EGFR: epidermal growth factor receptor; HER1: human epidermal growth factor receptor type 1; HER2: human epidermal growth factor receptor type 2; ICG: Indocyanine Green; IHC: immunohistochemistry; mAb: monoclonal antibody; NIR: near infrared; ON: always on; Pan: panitumumab; PBS: phosphate-buffered saline; SDS: sodium dodecyl sulfate; SQ: self-quenched; Tra: trastuzumab.

## Competing interests

The authors declare that they have no competing interests.

## Authors' contributions

KS conducted experiments, performed analysis and wrote the manuscript. MM and TN conducted experiments and performed analysis. PLC wrote the manuscript and supervised the project, while HK planned and initiated the project, designed and conducted experiments, wrote the manuscript, and supervised the entire project. All authors read and approved the final manuscript.
